# Immunotherapy Potentiates the Effect of Chemotherapy in Metastatic Melanoma—A Retrospective Study

**DOI:** 10.3389/fonc.2020.00070

**Published:** 2020-02-14

**Authors:** Reut Hadash-Bengad, Emma Hajaj, Shiri Klein, Sharon Merims, Stephen Frank, Galit Eisenberg, Alexander Yakobson, Marina Orevi, Nadia Caplan, Tamar Peretz, Michal Lotem, Jonatan E. Cohen

**Affiliations:** ^1^Sharett Institute of Oncology, Hadassah Medical Center, The Faculty of Medicine, Hebrew University of Jerusalem, Jerusalem, Israel; ^2^Department of Oncology, Soroka University Medical Center and Faculty of Health Sciences, Ben Gurion University of the Negev, Be'er Sheva, Israel; ^3^Department of Nuclear Medicine, Hadassah Medical Center, The Faculty of Medicine, Hebrew University of Jerusalem, Jerusalem, Israel; ^4^Division of Radiology and Medical Imaging, Hadassah Medical Center, The Faculty of Medicine, Hebrew University of Jerusalem, Jerusalem, Israel; ^5^The Wohl Institute for Translational Medicine, Hadassah Medical Center, Jerusalem, Israel

**Keywords:** immune checkpoint inhibitors, malignant melanoma, chemotherapy, salvage therapy, immune-monitoring

## Abstract

Melanoma survival increased with targeted- and immunotherapy agents, yet most patients ultimately progress and require salvage therapy. In our experience, some progressive disease patients on immune-checkpoint inhibitors (ICIs) demonstrate deep and sustained responses to chemotherapy. We hypothesized that ICIs improve the response to subsequent chemotherapy in metastatic melanoma. We conducted a retrospective study to compare the efficacy of chemotherapy given with prior immunotherapy, to its efficacy given without it. We measured progression free survival (PFS), overall survival, and response rate. Immune-monitoring was performed on sequential peripheral blood mononuclear cell samples taken from a chemotherapy-responsive patient. The chemotherapy post-immunotherapy group (CpI) included 11 patients, the chemotherapy without prior immunotherapy (CNPI) group included 24 patients. Median PFS was 5.2 months in the CpI vs. 2.5 months in the CNPI groups; HR 0.37 [95% Confidence interval (CI) 0.144–0.983], *P* = 0.046. Immune-monitoring showed an increased proportion of CD8^+^ cells, with elevated PD-1 and CD69 expression, while on chemotherapy, as compared with all-time points on ICIs, suggesting immune-activation. Immunotherapy potentiates the effect of chemotherapy in metastatic melanoma possibly through activation of CD8^+^ T cells.

## Introduction

Metastatic melanoma has evolved in recent years from an almost universally lethal disease to one with effective treatments and potential of a cure. The two major therapeutic advances were the development of targeted therapy toward the BRAF mutation-driven, constitutively-activated MAPK pathway using inhibitors of BRAF and MEK, and immunotherapy which used immune checkpoint inhibitors (i.e., anti-CTLA4 and anti-PD1 monoclonal antibodies). Indeed, median overall survival has increased from ~9 months in the chemotherapy era (1), to almost 24 months with a combination of the BRAF and MEK inhibitors dabrafenib and trametinib (2), and 37.5 months with the anti-PD-1 nivolumab (3). Even more important is the large fraction of long term survivors, with early reports of 41% 5-year survival rates with first line anti-PD1 (4) and a 5-year survival rate of 52% with combination checkpoint inhibition (5).

Nonetheless, a large fraction of patients will demonstrate primary- or develop acquired resistance to these therapies (6). Some of the progressing patients will still be fit for further lines of therapy and thus may be offered chemotherapy.

In fact, until the newly developed therapies, chemotherapy was the mainstay of treatment for metastatic patients. Dacarbazine (DTIC), an alkylating agent, was approved in 1975 by the FDA for the treatment of metastatic melanoma. The approval was granted solely on the basis of response rate data, with no survival advantage shown. Response rate in early trials of DTIC was ~20% (7), and in data from recent years, when DTIC was used in the control arm of BRAF and PD-1 inhibitor trials, response rate was recorded at 14% (8). Other single and combination chemotherapy regimens, for example BOLD (bleomycin, vincristine, lomustine, DTIC) and CVD (cisplatin, vinblastine, DTIC) were tested over the years, still not demonstrating a survival benefit (9, 10). In light of these non-impressive data, the question arises as to whether there is a role for chemotherapy in current melanoma practice.

The basic mechanism by which chemotherapy induces tumor regression is direct cytotoxicity. However, this class of drugs has additional effects that change the immune milieu of the tumor. For example, cyclophosphamide and gemcitabine preferentially deplete T regulatory cells (Treg), while paclitaxel and 5-fluorouracil inhibit myeloid-derived suppressor cells [MDSCs; Reviewed by Hellmann et al. (11)]. As such, chemotherapy may interact with cancer immunotherapy to facilitate a superior immune-response. Indeed, based on this rationale, recent trials have tested *combinations* of chemotherapy with immune checkpoint inhibitors. For example, the Keynote 189 study randomized patients with non-small cell lung cancer in a phase III study of platinum and pemetrexed with or without pembrolizumab. The combination showed an improved 12-month overall survival of 69.2%, compared with 49.4% on chemotherapy alone. This indicated at least an additive effect of the combination, but didn't substantiate a synergistic effect (12).

A phase II randomized study assessed the safety and efficacy of ipilimumab both alone and in combination with DTIC across 72 patients with metastatic melanoma. While results were not significant, there was a numerically-greater objective response rate and disease control rate in the ipilimumab-with-DTIC group compared with the ipilimumab-alone cohort (13).

The phenomenon of improved response to chemotherapy when administered *after* immunotherapy has recently been described retrospectively in two series on non-small cell lung cancer (NSCLC) and squamous cell carcinoma of the head and neck, as well as a small mixed histology cohort (14–17). The biological mechanism of these findings was not explored in these reports. Our own clinical experience has seen us observe patients who had progressive disease on immune checkpoint inhibitors and experienced significant tumor shrinkage when switched to subsequent chemotherapy. Could there be a priming effect of immune checkpoint inhibitors, which later leads to unprecedented responses to chemotherapy, of a quality surpassing what was seen in the past?

Based on our observations and data pertaining to chemotherapy modulation of the tumor-microenvironment, we hypothesized that previous treatment with immune checkpoint inhibitors increases the efficacy of chemotherapy when given at a later stage of the disease, in patients with metastatic melanoma.

In this single-center retrospective study, we aimed to evaluate the efficacy of chemotherapy administered in the setting of metastatic melanoma after previous treatment with immune-checkpoint inhibitors, compared to chemotherapy administered to similar patients who had not received prior immune-checkpoint inhibitors. In the experimental arm of the study, a longitudinal immune evaluation of lymphocyte activation and exhaustion markers was undertaken on one patient to explore possible immune-mechanisms involved.

## Materials and Methods

This was a single-center retrospective study carried out at the Sharett Institute of Oncology at Hadassah Medical Center. Patient data retrieval from electronic files was approved by the institutional review board (Hadassah Medical Organization IRB, Approval number 0306-16-HMO). Patients were included if they had histologically confirmed diagnosis of stage IV malignant melanoma and had received chemotherapy as part of their therapy, irrespective of therapy line. The experimental arm comprised patients who received chemotherapy following immune checkpoint inhibition (ICI) treatment with anti PD-1, anti CTLA-4 alone, or in combination. In the control group, patients were treated with chemotherapy as first line, or second line after targeted therapy. Patients were excluded if they (1) had a diagnosis of uveal or acral melanoma, (2) received chemotherapy solely as a radio-sensitizer.

Since no statistically significant survival improvement with bio-chemotherapy over chemotherapy was reported (18), bio-chemotherapy regimens including interleukin-2 (IL-2) were considered “chemotherapy” despite the immunotherapeutic benefit attributed to IL-2.

OS and PFS were measured from chemotherapy initiation to the date of death or disease progression, and summarized descriptively using the Kaplan Meier survival analysis and log rank test to compare the groups. We used the Cox model for hazard ratio (HR), confidence intervals of 95%, α level of 0.05.

If a patient received multiple lines of chemotherapy, analysis was performed on the first chemotherapy line.

Complete response (CR) was defined as the resolution of all imaging evidence of disease; partial response (PR) as a decrease in the size of a tumor in response to treatment; mixed response (MR) as a decrease in size of at least one of the lesions and an increase in size of at least one of the others; stable disease (SD) as the absence of change in size in two sequential imaging tests; and progressive disease (PD) as the increase in size of at least one of the lesions.

All statistical analyses were performed using SPSS software version 21.0. Univariate analysis was carried out for PFS, OS, and response.

### Peripheral Blood Mononuclear Cell Analysis

Blood samples were drawn at several time points during treatment. Peripheral blood mononuclear cells (PBMCs) were purified, washed and counted. 1 × 10^5^ Lymphocytes in each sample were activated with plate bound anti CD3 (1.0 μg/ml, clone OKT3, Invitrogen). The cells were than stained with the following antibodies: anti- CD8 PB (clone RPA-T8, BioLegend), anti PD-1 APC (clone EH12.2H7, BioLegend), anti CD69 APC (clone FN50, BioLegend).

### Flow Cytometry

Assays were performed using CytoFlex (Beckman Coulter). Flow cytometry analysis was done using FCS Express 5 Flow research edition (De Novo software).

## Results

### Case Studies

**Figure 1:**
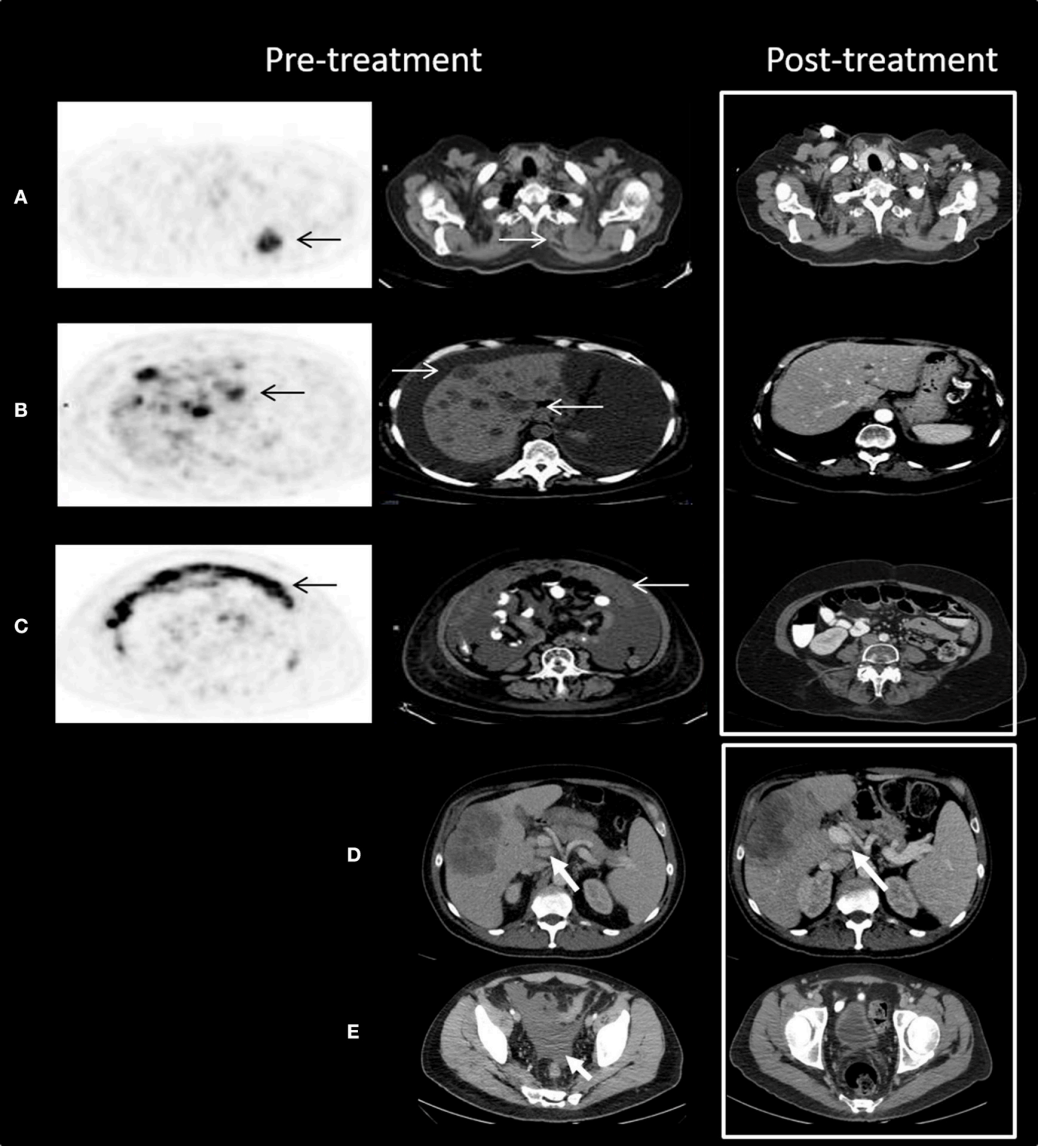
Outstanding response to chemotherapy after progression on anti-PD1 treatment. **(A–C)** Patient R.H. Trans-axial slices of pre-treatment ^18^F-FDG PET/CT scan illustrate advanced metabolically highly active (black arrows) metastatic disease in the following areas: **(A)** the left upper chest posterior 4.0 cm mass (white arrows) with SUV max 11.3; **(B)** multiple liver lesions with SUV max up to 15.8; and **(C)** omental cake with SUV max up to 14.2. Post-treatment CT scan (white frame) demonstrates complete resolution of all pre-treatment findings. **(D,E)** Patient M.G. Axial slices of contrast enhanced CT scan show enlarged porta hepatis lymph nodes **(D)** and medium amount of ascites in the pelvis **(E)**. Following treatment, the lymph nodes decreased in size and the ascites resolved. Decrease in size of the metastatic deposit in the right hepatic lobe can be also seen.

Two patients are described as having significant responses to chemotherapy-as-salvage after progressing on ICIs, drawing our attention to this phenomenon. The first, R.H., a 64-year-old patient, was diagnosed with vulvar melanoma that did not respond to cisplatin, vinblastine, and dacarbazine (CVD). She had a 2-month relief on pelvic irradiation, and during a course of five doses of anti-PD1 developed massive disease involving the liver and peritoneal cavity. She was a near-dying patient, requiring daily paracentesis. At this point she was started on a weekly protocol of paclitaxel and carboplatin. To our surprise, the patient's status improved significantly. After 8 courses of chemotherapy she was well-enough to be given a full course of ipilimumab. The patient achieved a complete remission of all metastatic deposits, which is now maintained for 17 months since the beginning of her chemotherapy. See Figures 1A–C for patient's imaging.

The second case, patient M.G., was a 51-year-old male with BRAF wildtype cutaneous melanoma, who developed liver metastases and was treated with ipilimumab with progressive disease. He was subsequently treated with pembrolizumab with a partial response as best response; ultimately progressing after nearly a year and a half. He then was treated with temozolomide and palliative liver radiation, resulting in a partial response with shrinkage of liver metastases, but also a good response outside the radiation field with shrinkage of liver hilum nodes, resolution of ascites, and stabilization of mesenterial and retroperitoneal nodes (see Figures 1D,E). This patient went on to receive adoptive cell therapy while still in response to temozolomide.

### Retrospective Cohort Study

A retrospective cohort study was undertaken to test our hypothesis that salvage chemotherapy after ICI would demonstrate a superior outcome as compared with chemotherapy with no prior ICI.

All patients included in this study had M1c\d disease (according to the AJCC 8th edition). The study group, i.e., chemotherapy post-immunotherapy (CpI), included 11 patients while the control group, i.e., chemotherapy with no prior immunotherapy (CNPI), included 24 patients. Chemotherapies received in the CpI group included dacarbazine, temozolomide, and a combination of paclitaxel with carboplatin; dacarbazine, temozolomide, cisplatin, bleomycin, paclitaxel, carboplatin, fotemustine, or carmustine in the CNPI group. Prior Immunotherapy in the CpI group included anti PD-1 (pembrolizumab or nivolumab), anti CTLA-4 (ipilimumab), or sequential treatment with both classes; some patients had also received targeted therapy. The mean time from immunotherapy cessation to chemotherapy initiation was 3.2 months (range: 0–9 months). None of the patients received chemotherapy or immune checkpoint inhibitors in the *adjuvant* setting. Some patients in the CNPI group continued treatment in other institutions, thus not all follow-up data was available. Subsequent treatments included anti PD-1 (pembrolizumab or nivolumab), anti CTLA-4 (ipilimumab), targeted therapy in the CNPI group, as well as adoptive cell transfer, letrozole, and anti CD20 in the CpI group. See Table 1 for patient characteristics.

**Table 1:** Patient characteristics.

	**CNPI**	**CpI**	***P*-value**
	**Chemotherapy as first line or second line after targeted therapy (*N* = 24)**	**Chemotherapy after immunotherapy (*N* = 11)**	
Median age (Range)	50 (25–79)	63 (46–83)	0.021
**Sex**			
Female	7 (29.2%)	3 (27.3%)	NS^*^
Male	17 (70.8%)	8 (72.7%)	
**AJCC 8 Stage at chemotherapy initiation (*****N*****, %)**			
M1c	11 (46%)	6 (55%)	NS^*^
M1d	13 (54%)	5 (45%)	
	*N* = 24	*N* = 11	
**Type of chemotherapy (*****N*****, %)**			
Single agent	12 (50%)	10 (91%)	
Combination chemotherapy	5 (20%)	1 (9%)	
Bio-chemotherapy^a^	7 (30%)	0	
**Previous lines of treatment (*****N*****, %)**			
Range	0–1	1–4	NA^**^
None	19 (79.1%)	0	
1	5 (20.8%)	2 (18.18%)	
2	0	7 (63.63%)	
3	0	0	
4	0	2 (18.18%)	
	*N* = 24	*N* = 11	
**Type of immunotherapy (*****N*****, %)**			
Anti PD-1	NA^**^	2 (18.18%)	
Ipilimumab		2 (18.18%)	
Sequential anti PD-1 AND ipilimumab		7 (63.63%)	
**Subsequent lines of treatment**			
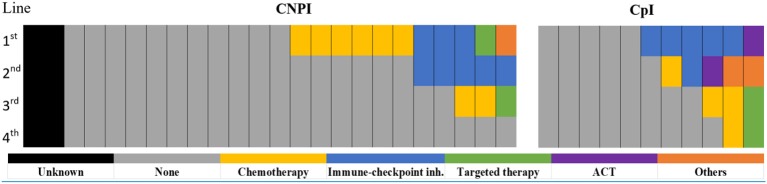			

* Not significant.

** Not applicable.

a *Bio-chemotherapy- chemotherapy + IL-2*.

#### Progression Free Survival

For PFS analysis there was data available from 28 patients, 11 in the CpI group, and 17 in the CNPI group (for the remaining, only survival data was available). For one patient in the CpI group, and 6 patients in the CNPI group, deterioration was rapid and progression was determined on the basis of clinical impression.

**Figure 2:**
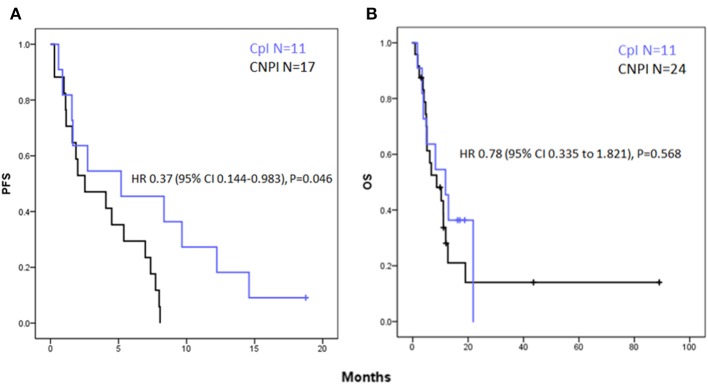
Kaplan- Meier curves for **(A)** progression free survival showing a significant increase in PFS in the CpI group vs. CNPI with a hazard ratio (HR) of 0.37; [95% Confidence interval (CI) 0.144–0.983], *P* = 0.046. **(B)** Overall survival showing a non-significant trend toward improved OS in the CpI group HR 0.78 [95% Confidence interval (CI) 0.335–1.821], *P* = 0.568.

Median progression-free survival was 5.2 and 2.5 months in the CpI and CNPI groups, respectively. Log Rank (Mantel-Cox) Test of equality of survival distributions: *P* = 0.039 (Figure 2A). HR for PFS associated with CpI as compared with CNPI for PFS was 0.37 [95% Confidence interval (CI) 0.144–0.983], *P* = 0.046.

#### Overall Survival (OS)

Median overall survival was 11.8 and 8.6 months in the CpI and CNPI groups, respectively. Log Rank (Mantel-Cox) test of equality of survival distributions: *P* = 0.566 (Figure 2B). HR associated with CpI as compared with CNPI for OS was 0.78 [95% Confidence interval (CI) 0.335–1.821], *P* = 0.568.

One-year survival was numerically higher in the CpI group with 46% (5/11) than the CNPI group with 21% (5/24), however the difference was not statistically significant (*P* = 0.138 using Fisher's exact test).

#### Response Rate (RR)

The RR in CpI was 36.4% (4/11) vs. 19.0% (4/21) in the CNPI group. *P* = 0.256 in Fisher's exact test.

### Immune-Monitoring

We analyzed PBMCs of patient M.G. (described above) to explore potential immune correlates of response to chemotherapy after immune checkpoint inhibition.

**Figure 3:**
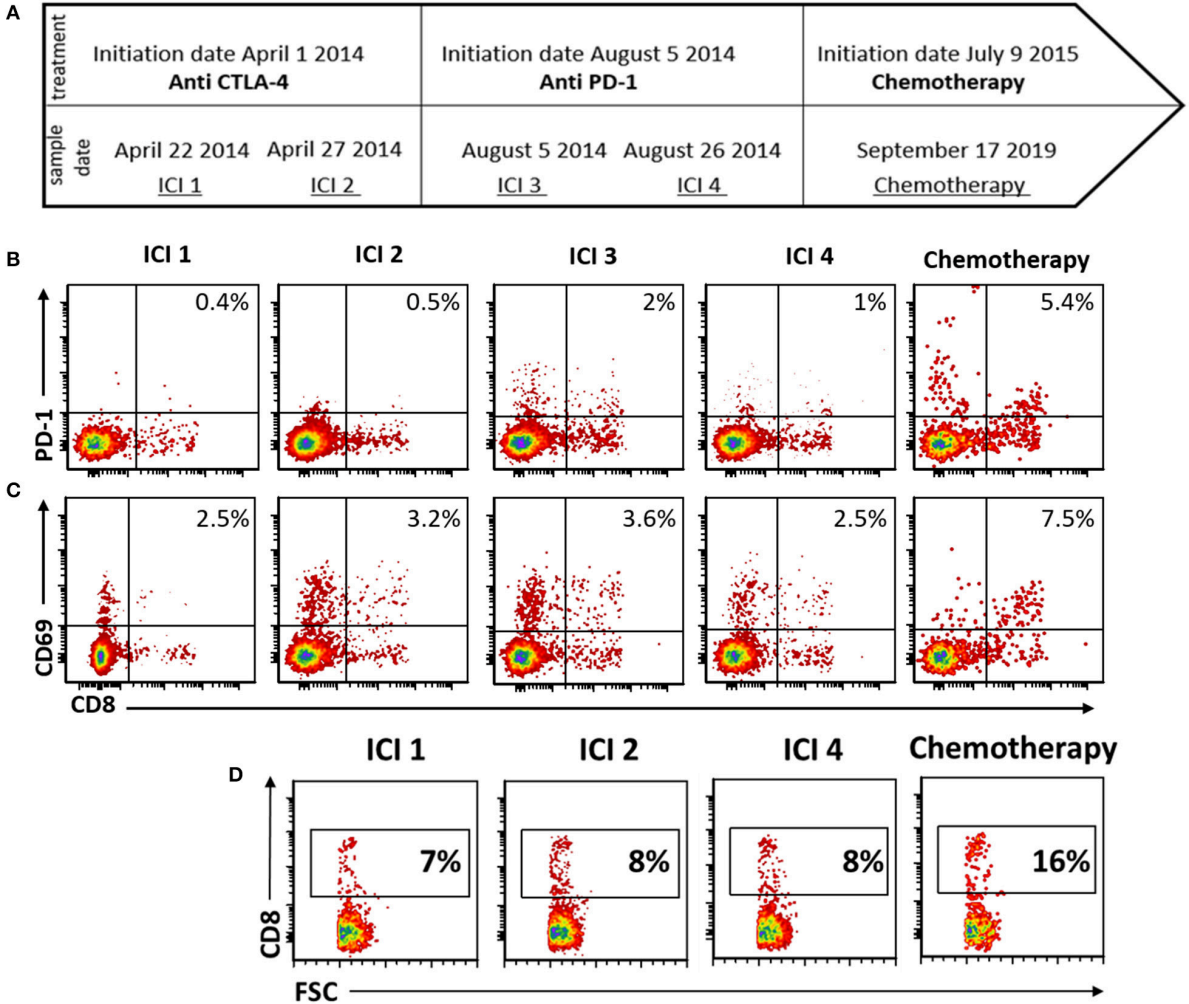
Peripheral blood immune-monitoring demonstrates an increase in effector phenotype and CD8+ fraction following chemotherapy. **(A)** Timeline of blood collection and corresponding treatment given. **(B–D)** Flow cytometry of peripheral mononuclear blood cells (PBMCs) following activation with anti CD3 **(B)** stained for PD-1. **(C)** CD69 and **(D)** CD3.

PBMCs from M.G. were analyzed for CD69 and PD1 expressions following overnight anti-CD3 activation. The activation step was included in order to reflect the functional capacity of the patient's lymphocytes. CD69 is a pure activation marker, while PD-1 is induced on activated T cells but also contributes to subsequent exhaustion (19, 20). Blood available for analysis included 4 time points during immune check-point inhibitor (ICI) treatment, and one time point during chemotherapy. ICI time points 1 and 2, drawn during progressive disease on ipilimumab treatment, taken 3 days apart; ICI time point 3 was drawn on the day of pembrolizumab initiation; and ICI time point 4 was drawn 20 days later (Figure 3A). The chemotherapy time point was drawn during a partial response to temozolomide.

During chemotherapy treatment, the activation marker PD-1 was increased on both CD8+ and CD8– T cells, as compared with all the time points during ICI treatment (Figure 3B). Expression of CD69 was increased as well on CD8 T cells during chemotherapy (Figure 3C). Furthermore, the proportion of CD8 cells from total PBMCs was increased during chemotherapy (Figure 3D).

## Discussion

The introduction of checkpoint inhibitors to the treatment of metastatic melanoma marked a turning point in a disease that was essentially incurable. The disappointment from chemotherapy regimens for melanoma discouraged the designing of clinical trials in which PD-1 inhibitors would be given in combination with chemotherapy. Thus, little data was gathered regarding the interplay between chemotherapy and immunotherapy.

Despite the impressive responses achieved with immune checkpoint inhibitors, over half of the patients will not respond or progress on these treatments, and many will receive another line of treatment. At this point, the role of chemotherapy as a late treatment option rises again as a question of major clinical significance. However, the paucity of data on this subject necessitates the retrieval of retrospective patient series in order to identify trends. This was the incentive for this study: to validate early single patient case-based impressions that immunotherapy improves the response to subsequent chemotherapy in metastatic melanoma.

In this series, comparing 11 melanoma patients who received chemotherapy after failing immunotherapy to 24 immunotherapy-naïve patients, we found a statistically significant improvement in PFS, and a trend toward improvement in OS and RR among the patients who had prior immunotherapy. Though not statistically significant, improvement in OS was clinically meaningful. Furthermore, we calculated OS from initiation of chemotherapy. However, in the CpI group this was an advanced line of therapy, under-representing OS from metastatic disease diagnosis, as opposed to the CNPI group for which OS is the true survival from stage IV diagnosis (this further emphasizes the strength of the trend we observed for OS). A recent single arm study from Japan described post-ICI responses to chemotherapy in 7 patients, with two achieving partial response and another two stable disease, lending further support to our findings (21).

The improved outcome of chemotherapy given after immunotherapy is even more remarkable considering that, in general, the benefit of treatment declines as a function of a line of therapy. In fact, 63.63% of the CpI group received chemotherapy as a third line, while in the CNPI group 81.81% received chemotherapy as their first line of treatment, and yet CpI had an improved PFS. Conversely, in the CpI group, a selection bias favoring patients with less aggressive disease must be considered, as those with aggressive disease may not have reached late lines of therapy.

Although chemotherapies were traditionally believed to be purely immunosuppressive, evidence emerged that when chemotherapy is given at the right dose and sequence, it may result in enhancement of the immune response. Chemotherapy can enhance the innate immune system via stimulating macrophages, natural killer (NK) and dendritic cells (DCs), as well as by disrupting immune suppressor mechanisms through eliminating myeloid-derived suppressor cells (MDSCs). It may also enhance the adaptive immune system by acting, among others, on T regulatory cells (22). Indeed, vaccination approaches toward cancer have incorporated low dose cyclophosphamide in order to augment the immune response (23). Our current findings are in-line with such data, supporting an important immune-activation role for chemotherapy in metastatic melanoma patients.

The immune monitoring results from one of the patients further support chemotherapy immune enhancement, illuminating the effect on cytotoxic T cells (CTLs). Chemotherapy, and specifically the alkylating agents temozolomide and dacarbazine commonly prescribed to our cohort, are known to induce lympho-depletion. Paradoxically, the proportion of CTLs in the peripheral blood of the patient we monitored *increased* upon receiving chemotherapy in the post-immunotherapy setting. We furthermore found an increase in the activation markers CD69 and PD-1, suggesting the CD8 cells are not only more numerous but are in a more active state. These preliminary data point to a possible mechanism for our observed clinical findings, by which T cells primed by prior immunotherapy are further activated following exposure to chemotherapy, leading to both direct cytotoxic tumoricidal effects alongside immune-dependent anti-neoplastic activity. This data is derived from a single patient and as such is highly exploratory, yet intriguing.

In this retrospective study we provide evidence for an immunological effect of chemotherapy in metastatic melanoma following immune checkpoint inhibition, associated with clinical efficacy surpassing historical (pre-checkpoint inhibition) data. These data support the continued use of chemotherapy in advanced lines in current practice, providing evidence that expected efficacy may be significantly higher than previously thought. Further investigations are required in order to validate these findings and establish the mechanism of the interaction between these two modalities. Better mechanistic understanding will provide the basis for intelligent planning of future combinations and sequencing studies.

## Data Availability Statement

The datasets generated for this study are available on request to the corresponding author.

## Ethics Statement

The studies involving human participants were reviewed and approved by Hadassah Medical Organization IRB. Written informed consent for participation was not required for this study in accordance with the national legislation and the institutional requirements. Written informed consent was not obtained from the individual(s) for the publication of any potentially identifiable images or data included in this article.

## Author Contributions

RH-B, SM, SF, GE, AY, TP, ML, and JC contributed to conception and design of the study. RH-B, SM, SF, ML, and JC collected the clinical data. Experiments conducted by RH-B, EH, SK, and GE. Clinical and imaging data analyzed by MO and NC. RH-B and JC performed the statistical analysis. RH-B, SK, ML, and JC wrote the first draft of the manuscript. All authors contributed to manuscript revision, read, and approved the submitted version.

### Conflict of Interest

The authors declare that the research was conducted in the absence of any commercial or financial relationships that could be construed as a potential conflict of interest.
